# Eating disorders and smartphone addiction among university students: Which relationship?

**DOI:** 10.1192/j.eurpsy.2023.1360

**Published:** 2023-07-19

**Authors:** M. Turki, H. E. Mhiri, F. Jmil, O. Elleuch, A. Samet, S. Ellouze, N. Halouani, J. Aloulou

**Affiliations:** PSYCHIATRY “B”, HEDI CHAKER UNIVERSITY HOSPITAL, SFAX, Tunisia

## Abstract

**Introduction:**

Nowadays, most of the university youth use smartphones resembling a mini-computer. Despite its benefits, it has been shown that smartphone use is associated with increased anxiety, insomnia, lack of self-confidence, emotional disturbances, as well as negative effects on energy level, body weight and eating habits.

**Objectives:**

This study aimed to assess the relationship between smartphone addiction and eating disorders in university students.

**Methods:**

It was a cross-sectional web-based study, conducted among 108 university students in Tunisia. Data were collected using an online questionnaire spread throughout social media (Facebook), using the Google Forms® platform. Eating disorders were assessed via “EATING ATTITUDES TEST-26” (EAT-26) and smartphone addiction via “SMARTPHONE ADDICTION SCALE-SHORT VERSION” (SAS-SV).

**Results:**

The mean age of participants was 22.11±2.2 years, with a sex-ration(F/M) of 3.7.

The mean score SAS-SV was 38.3. Among the students, 75.9% were considered at high risk of Smartphone addiction.

The mean score EAT-26 was 20.45. A problematic eating behavior was noted in 32.4% of participants.

Students with higher SAS-SV scores were more likely to have higher EAT-26 scores (p=0.013; r=0.237). Students being at risk of eating disorder were found to have higher SAS-SV scores (40.9 vs 37; p=0.033).

**Conclusions:**

Our findings suggested that smartphone addiction seems to be associated with the development of eating disorders. Thus, university students should be encouraged to join social communities so that they may take a break from technology and spend their leisure time developing meaningful relationships.

**Disclosure of Interest:**

None Declared

